# Mechanisms of Nausea and Vomiting: Current Knowledge and Recent Advances in Intracellular Emetic Signaling Systems

**DOI:** 10.3390/ijms22115797

**Published:** 2021-05-28

**Authors:** Weixia Zhong, Omar Shahbaz, Garrett Teskey, Abrianna Beever, Nala Kachour, Vishwanath Venketaraman, Nissar A. Darmani

**Affiliations:** 1 Department of Basic Medical Sciences, College of Osteopathic Medicine of the Pacific, Western University of Health Sciences, 309 East Second Street, Pomona, CA 91766, USA; wzhong@westernu.edu (W.Z.); gteskey@westernu.edu (G.T.); vvenketaraman@westernu.edu (V.V.); 2 School of Medicine, Universidad Iberoamericana, Av. Francia 129, Santo Domingo 10203, Dominican Republic; omarshahbaz1122@gmail.com; 3 Graduate College of Biomedical Sciences, Western University of Health Sciences, Pomona, CA 91766, USA; abrianna.zorzi@westernu.edu (A.B.); nala.kachour@westernu.edu (N.K.)

**Keywords:** nausea, vomiting, emesis, G-protein coupled receptors, signaling pathway, Ca^2+^, brainstem, the gastrointestinal tract

## Abstract

Nausea and vomiting are common gastrointestinal complaints that can be triggered by diverse emetic stimuli through central and/or peripheral nervous systems. Both nausea and vomiting are considered as defense mechanisms when threatening toxins/drugs/bacteria/viruses/fungi enter the body either via the enteral (e.g., the gastrointestinal tract) or parenteral routes, including the blood, skin, and respiratory systems. While vomiting is the act of forceful removal of gastrointestinal contents, nausea is believed to be a subjective sensation that is more difficult to study in nonhuman species. In this review, the authors discuss the anatomical structures, neurotransmitters/mediators, and corresponding receptors, as well as intracellular emetic signaling pathways involved in the processes of nausea and vomiting in diverse animal models as well as humans. While blockade of emetic receptors in the prevention of vomiting is fairly well understood, the potential of new classes of antiemetics altering postreceptor signal transduction mechanisms is currently evolving, which is also reviewed. Finally, future directions within the field will be discussed in terms of important questions that remain to be resolved and advances in technology that may help provide potential answers.

## 1. Introduction

Nausea and vomiting are essential protective defense processes by which humans as well as vomit-competent animals tend to avoid ingestion and/or digestion of potentially toxic substances. Nausea is the unpleasant sensation of having the urge to vomit, whereas vomiting (emesis) is a physical event and is the forceful expulsion of intestinal and gastric contents through the mouth [1]. Vomiting is often preceded by retching, where the content of gastrointestinal tract is forced into the esophagus, without expulsion of the vomitus [1]. Oftentimes, nausea and vomiting occur on a temporal continuum, but this is not always the case. Sometimes, severe nausea can be present without emesis, and, less frequently, vomiting may be present without nausea. Thus, nausea is no longer considered only a penultimate step of vomiting [2].

**Figure 1:**
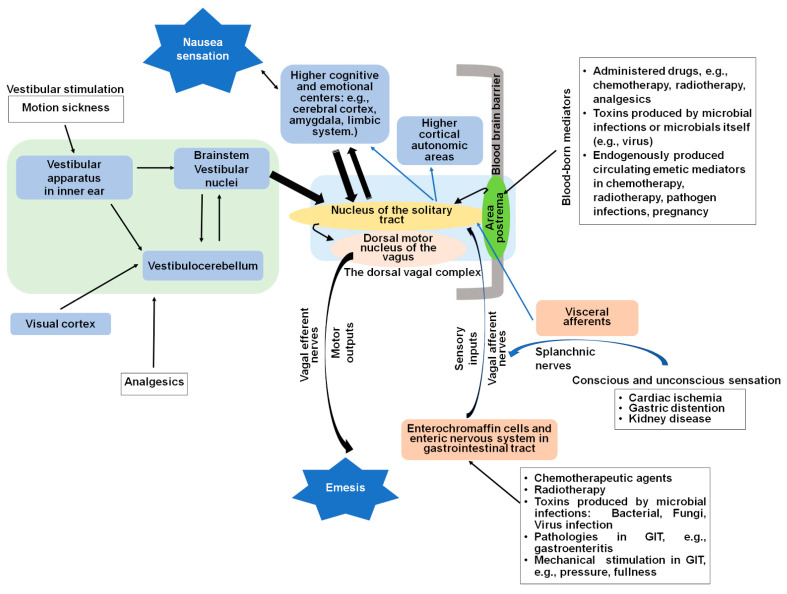
Central and peripheral anatomical sites involved in nausea and vomiting induced by various stimuli. Nausea and vomiting can be generated by diverse stimuli and are mediated by the bidirectional interaction between brain and gut. In brief: (1) The brainstem area postrema in the floor of the fourth ventricle lacks blood brain barrier and thus serves as direct central receptor sites for circulating and systemic emetic stimuli in the cerebrospinal fluid and the blood [11]. (2) Systemically administered drugs can activate corresponding receptors present on vagal afferents, which project sensory signals to the nucleus of the solitary tract [11,12]. (3) Peripheral stimuli such as toxic drugs and microbials (e.g., bacteria, viruses, fungi) that enter the lumen of the gastrointestinal tract (GIT) and pathologies in the GIT cause release of local emetic neurotransmitters/modulators, which subsequently act on the corresponding receptors present on vagal afferents and/or stimulate the brainstem area postrema via circulating blood [9,10]. Besides the area postrema and the sensory vagal afferents, the nucleus of the solitary tract is also the recipient of: (i) direct neural inputs from the splanchnic nerves carrying sensation caused by diseases of visceral organs (e.g., cardiac, kidney); (ii) brainstem vestibular nuclei collecting signals from vestibular apparatus in inner ear and/or cerebellum, caused by stimuli related to motion sickness and opioid analgesics [13,14]; and (iii) the cerebral cortex and limbic system, which accept and process emotional and cognitive stimuli [3,4,5,6,7,8]. The nucleus of the solitary tract has output pathways to the dorsal motor nucleus of the vagus, which further project to the upper gastrointestinal tract to produce the vomiting reflex [11]. In addition, the nucleus of the solitary tract has projections to the mid- and forebrain for the perception of nausea [15].

Nausea and vomiting can be triggered by several mechanisms, as depicted in Figure 1, including: (i) toxins/drugs/bacteria/viruses/fungi that enter the lumen of the gastrointestinal and, subsequently, indirectly stimulate the brainstem emetic nuclei located in the dorsal vagal complex via release of local emetic neurotransmitters in the upper gastrointestinal tract and subsequent activation of corresponding receptors present on vagus nerves and/or splanchnic nerves; (ii) toxic agents/drugs or infectious organisms that enter the body systemically and that may act directly on the dorsal vagal complex emetic nuclei in the brainstem; (iii) pathologies in the gastrointestinal tract, which stimulate vagal afferents or other visceral organs (e.g., cardiac), which stimulate visceral afferents; (iv) emotional and cognitive stimuli within the central nervous system (CNS), including cerebral cortex and the limbic system; and (v) disturbances of the vestibular nuclei and cerebellum in motion sickness [3,4,5,6,7,8]. The processes of nausea and vomiting result from continuous interactions between the gastrointestinal tract, including its enteric nervous system, the CNS, and the autonomic nervous system [9,10].

In this article, we will initially describe the underlying neuroanatomical, neurochemical, and receptor basis of diverse causes of nausea and vomiting along with corresponding, currently available agonist/antagonist antiemetics, followed by their potential intracellular signaling cascades. Finally, we will discuss the potential future treatments for nausea and vomiting caused by different stimuli. Understanding the circuitry and cellular mechanisms of nausea and vomiting helps us in developing new medications for the prevention and treatment of these debilitating symptoms. 

## 2. Central and Peripheral Sites of Nausea and Vomiting

The gut–brain axis is a continuous, bidirectional neural and endocrine communication network between the gastrointestinal tract and the brain that is crucial to gastrointestinal regulation [16]. The gut–brain axis includes the gastrointestinal tract and the afferent and efferent vagal nerves and spinal cord, the brainstem, limbic and interoceptive regions, and autonomous system and motor outputs [11]. This axis plays an important role in the initiation and maintenance of nausea and vomiting caused by diverse emetic stimuli. The central and peripheral compartments [17,18] of the emetic reflex arc are shown in Figure 1. The key sites in the mediation of vomiting are: (i) the brainstem dorsal vagal complex emetic nuclei such as the area postrema (AP), the nucleus of the solitary tract, and the dorsal motor nucleus of the vagus (DMNV) and (ii) the peripheral sites, including the sentinel epithelial enterochromaffin cells, which are present in the mucosa of gastrointestinal tract; the enteric nervous system embedded in the wall of gastrointestinal tract; and the vagus and splanchnic nerves. These peripheral emetic loci transfer emetic signals between the gastrointestinal tract as well as other internal organs, to the dorsal vagal complex emetic nuclei in the brainstem [5]. 

### 2.1. The Dorsal Vagal Complex

Different stimuli initiate the emetic reflex universally coordinated by the dorsal vagal complex in the brainstem, which is composed of the area postrema, nucleus of the solitary tract, and dorsal motor nucleus of the vagus [19]. The area postrema in the floor of the fourth ventricle as well as the nucleus of the solitary tract lack blood brain barrier, making neurons in these areas respond relatively rapidly to circulating and systemic stimuli in the blood and cerebrospinal fluid, which can trigger nausea and vomiting, and therefore serve as major central receptor sites for peripheral emetic stimulation [11]. The nucleus of the solitary tract is also the recipient of direct neural inputs from the area postrema and the sensory vagal afferents, as well as direct or indirect inputs from the splanchnic nerves, vestibular apparatus in inner ear, cerebellum, and the cerebral cortex, all of which play roles in the regulation of medullary reflexes controlling nausea and vomiting [3,5,6,7,8,18,20]. The nucleus of the solitary tract, and specifically its medial subnucleus, is the key integrative site for CNS modulation of the emetic reflex [17,18]. Via the nucleus of the solitary tract, the sensory signals are sent to the dorsal motor nucleus of the vagus, which mediates the emetic motor function of the gastrointestinal tract in the process of vomiting [9,17,21]. The dorsal motor nucleus of the vagus contains preganglionic parasympathetic motoneurons [21]. Most dorsal motor nuclei of the vagus efferent vagal motor and sympathetic fibers project to the upper gastrointestinal tract and influence gastrointestinal tract motility [11]. Under normal conditions, the activity of dorsal motor nucleus of the vagus neurons that innervate the gastrointestinal tract is controlled by a tonic inhibitory input from the nucleus of the solitary tract [21]. In the case of emesis, cholinergic dorsal motor nucleus of the vagus neurons through vagal efferent pathways activate nicotinic acetylcholine receptors present on postganglionic neurons within the stomach and upper gastrointestinal tract [18].

### 2.2. Vagal Afferent Pathways Are Involved in Detection of Emetic Stimuli

Vagal afferent pathways are major peripheral emetic nerves that prominently participate in vomiting in response to emetic stimuli in the gastrointestinal tract [22]. Indeed, in response to peripheral emetic stimuli, gastrointestinal vagal afferent fibers carry the information regarding the physiological state of the gastrointestinal tract to the dorsal vagal complex, and activation of vagal afferents is involved in the generation of vomiting [18]. In fact, electrical stimulation of abdominal vagal afferents is capable of inducing emesis, and abdominal vagotomy suppresses vomiting evoked by the chemotherapeutic agent cisplatin [23]. 

### 2.3. The Gastrointestinal Tract

In the gastrointestinal tract, enterochromaffin cells synthesize > 90% of the body’s 5-hydroxytryptamine (serotonin, 5-HT), as well as large amounts of substance P (SP), which are both fundamental to gastrointestinal motility, nausea, and vomiting [24,25]. Chemical, mechanical, or neurological emetogenic stimuli induce 5-HT, and/or SP release from enterochromaffin cells in a calcium (Ca^2+^)-dependent manner [18,26,27]. Following their release, 5-HT, and probably SP, stimulate their corresponding emetic receptors (serotonin 5-HT_3_ and substance P neurokinin NK_1_ receptors, respectively), which are present on the vagal afferents initiate nausea and vomiting [28]. Since 5-HT in the bloodstream will be ionized at physiological pH, it is unlikely to reach the brainstem emetic nuclei; however, an active transport mechanism for substance P is present in the brainstem [26,27]. In fact, intraperitoneally administered substance P can enter the brainstem but not the frontal cortex [29]. Neurons in the enteric nervous system include the myenteric and submucosal plexuses, sensory and motor neurons, and interneurons [30]. While the exact function of various types of neurons in the gut enteric nervous system remains unclear, some studies support that damage to enteric neurons, including neuronal loss and morphological alterations, may underlie cancer chemotherapy-induced nausea and vomiting [31,32,33].

### 2.4. Nausea Studies: Models, Progress, and Limitations

Currently, significant knowledge exists on the neuroanatomy and neurochemistry of vomiting. Despite being closely associated, central pathways of nausea and vomiting appear to be at least partly different [2]. Our current understanding of central pathways of nausea has improved but remains very basic. Emesis is controlled in the brainstem, but the sensation of nausea is thought to arise from activation of higher levels of the CNS (e.g., the cerebral cortex) involved in conscious perception [19,34]. Projections from the nucleus of the solitary tract in the brainstem dorsal vagal complex or sensory inputs from the vestibular and cerebellum systems regarding body balance and position may stimulate the cortical nausea center [3]. Neurotransmitters and hormones involved in the sensation of nausea are diverse, and there is evidence that noradrenaline, 5-HT, vasopressin, substance P, cortisol, and the nonapeptide oxytocin [35] have some role in the genesis of nausea [19]. 

#### 2.4.1. Human Nausea Studies

Brain imaging as a biomarker of nausea has facilitated noninvasive identification of the activation sequence of brain structures involved in the nausea pathway [36]. The latter authors studied human volunteers experiencing motion sickness evoked by an optokinetic drum and employed functional magnetic resonance imaging (fMRI) to record self-reported nausea scores and suggest the brain regions involved in the evolution of nausea are numerous [36]. The medial prefrontal cortex and the pregenual anterior cingulate cortex, as well as areas of the brain involved in higher cognitive function and emotion, are found to be positively correlated with an increase in heart rate during nausea, suggesting the importance of cognitive and emotional centers in modulating the parasympathetic to sympathetic shift associated with nausea. Increased activity was also recorded in the left amygdala, the ventral putamen, and the putative locus coeruleus prior to the subject reporting of increasing nausea score, indicating these areas translate nauseogenic stimulus into fear conditioning and emotional triggering, which ultimately leads to the sensation of strong nausea. Increased nausea was also associated with continued and sustained increased activity in the following cortical regions: insula, cingulate, somatosensory, orbitofrontal, prefrontal, and premotor cortices and in subcortical structures, including the putamen, nucleus accumbens, and ventral tegmental area. These areas are involved in the interoceptive, limbic, somatosensory, and cognitive network. However, functional imaging technology is not widely accessible for veterinary species, and the utility of fMRI in animals is limited by the fact that it must be carried out in conscious, nonsedated subjects [19].

#### 2.4.2. Nausea Models in Non-Emetic Species

Several established animal models are the mainstay of preclinical nausea and vomiting research in both academia and pharmaceutical industry, including the dog, cat, ferret, house musk shrew [37], and least shrew [38]. Use of animal emesis models has allowed significant inroads in our understanding of the anatomy, neuronal pathways, and receptor subtypes, as well as their intracellular signals involved in vomiting evoked by diverse mechanisms both within the central nervous system and the periphery. 

In non-emetic animals, particularly rodents that lack a vomiting reflex and the capacity to vomit, behaviors such as pica (eating non-nutritive substances such as kaolin (also known as “white dirt”, “chalk”, or “white clay”)) and gaping can accompany or follow the response to emetic stimuli (i.e., cisplatin, copper sulfate, radiation) [39]. Pica and gaping behaviors are regarded as indices of nausea in rats and mice [37] and have been used to identify agents that have antiemetic activity [40,41,42,43]. Chemotherapy patients report anticipatory nausea and vomiting upon re-exposure to the cues previously associated with the treatment [44]. Thus, this animal model of conditioned disgust appears to closely parallel the anticipatory nausea experienced by many patients receiving cancer chemotherapeutics [35]. The conditioned gaping reaction has also been used as a preclinical tool to evaluate drug side effects such as nausea and emesis that might result from newly developed pharmaceutical agents [45,46]. 

## 3. Diverse Stimuli Evoke Vomiting via Distinct Receptors

**Figure 2:**
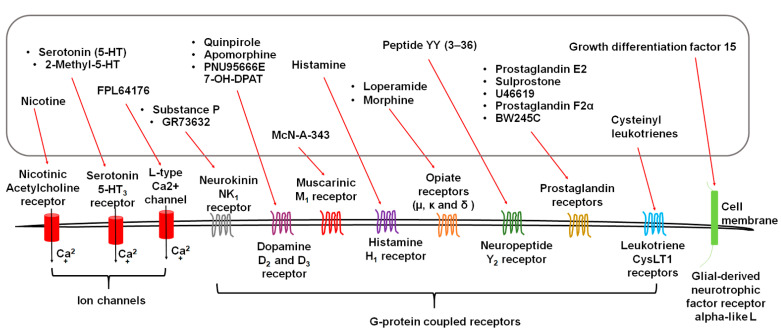
A schematic representation of emetic ligands or agonists, acting on the corresponding cell membrane-located receptors, including calcium-permeable ion channels and G-protein coupled receptors.

Exogenous stimuli, including emetogenic cancer chemotherapy, bacterial toxins in the gut, viruses and fungi infection, food poisoning, epigastric radiation, diverse drugs, and motion, evoke vomiting, which is frequently, but not always, accompanied by nausea [11]. They act either directly on their target site(s), or indirectly via release of emetic neurotransmitters/mediators, to activate corresponding emetic receptors (Figure 2), including serotonin type 3 (5-HT_3_), neurokinin 1 (NK_1_), dopamine D_2_ and D_3_, opioid mu and kappa, muscarinic M_1_, and histamine H_1,_ many of which are located both in the periphery (e.g., the gastrointestinal tract, vagal afferents) and the brainstem dorsal vagal complex (e.g., the area postrema) [11,12]. Per our discussion below, except for some ion channel coupled receptors such as 5-HT_3_ and transient receptor potential vanilloid type 1 receptor (TRPV1), many emetic receptors belong to the G-protein-coupled family of receptors (GPCRs).

### 3.1. Serotonin Receptors

The notion that 5-hydroxytryptamine (5-HT; serotonin) is involved in vomiting was deduced from drugs that interfere with its synthesis, storage, release, and metabolism long before the discovery of selective tools that modulate specific serotonergic receptor subtypes [47]. Serotonergic receptors can be classified into seven major families (5-HT_1–7_), which are trans-membrane G-protein-coupled receptors, except for the 5-HT_3_ receptor, belonging to the cys-loop ligand-gated ion channel family. The 7 families are divided further into subfamilies, and to date a total of 14 5-HT receptor subtypes have been described. Although basic evidence indicate that serotonin 5-HT_1–4_ receptors could be involved in the modulation of the emetic reflex, to date application of clinical evidence support a major emetic role for 5-HT_3_ receptors [47]. Being an ion channel, 5-HT_3_ receptor activation induces fast excitatory postsynaptic potentials and rapid depolarization of serotonergic neurons [48], leading to augmentation of intracellular Ca^2+^ concentration that causes the release of different emetic neurotransmitters and/or peptides (e.g., dopamine, cholecystokinin, glutamate, acetylcholine, substance P, or 5-HT, itself [49]. Significant clinical evidence indicates that both the first (e.g., ondansetron, Granisetron, dolasetron) and second (e.g., palonosetron) generation 5-HT_3_ receptor antagonists attenuate the first phase of cancer chemotherapy-evoked vomiting where serotonin is a major emetic player [50,51]. In fact, combination of a 5-HT_3_ receptor antagonist with a substance P neurokinin NK_1_ receptor antagonist plus dexamethasone is required to prevent both phases of highly emetogenic chemotherapy-evoked vomiting in approximately 90% of cancer patients. Although 5-HT_3_ receptor antagonists are generally considered as narrow-spectrum antiemetics, they are also useful for the suppression of postoperative nausea and vomiting, as well as pregnancy-induced emesis [50,51,52]. 

### 3.2. Substance P Neurokinin NK_1_ Receptor (NK_1_R)

Substance P is one member of structurally related mammalian family of tachykinin peptides, including neurokinin A, neurokinin B, and the N-terminally extended forms of neurokinin A such as neuropeptide K and neurokinin g [53]. Mammalian tachykinins activate three specific membrane-associated neurokinin receptors known as NK_1_, NK_2_, and NK_3_, which belong to the superfamily of G protein-coupled receptors. These receptors are recognized with moderate selectivity by endogenous substance P, neurokinin A, and neurokinin B, with respective preferential selectivity for NK_1_, NK_2_, and NK_3_ receptors. Although their affinity differences are not large and cross-interaction can occur across neurokinin receptors, substance P appears to be the endogenous ligand for NK_1_ receptors and has a major role in the induction of vomiting, particularly during the delayed phase of cancer chemotherapy-evoked emesis [17,18]. In fact, low doses of substance P (0.03–0.2 mg/kg) can evoke vomiting in dogs when administered intravenously [54] but not when injected intraperitoneally [55]. In fact, very large doses of intraperitoneally (i.p.) administered substance P (e.g., 50–100 mg/kg) are required to evoke vomiting in least shrews [29]. This is probably due to rapid metabolism of substance P following its intraperitoneal administration. In fact, administration of low doses of the selective NK_1_ receptor agonist GR73632 (1–5 mg/kg, i.p.) causes vomiting in least shrews in a dose-dependent fashion [29]. Both neurokinin NK_1_Rs and substance P are expressed in central and peripheral emetic loci, including the brainstem dorsal vagal complex emetic nuclei, as well as vagal afferents, enteric neurons, and enterochromaffin cells of the gastrointestinal tract [17,56]. Moreover, stimulation of NK_1_Rs by substance P excites neurons in the area postrema of dogs and subsequently evokes vomiting [57], whereas specific ablation of either intestinal or brainstem NK_1_Rs leads to blockade of vomiting evoked by the NK_1_R selective agonist GR73632 in the least shrew [29]. In fact, GR73632 is a potent emetogen in the least shrews, where central NK_1_Rs play a major role, and peripheral NK_1_Rs play a minor role, in the induction of vomiting [58]. In the clinic, NK_1_R antagonists (such as Aprepitant, netupitant and rolapitant) are recommended for the suppression of the second phase of vomiting evoked by highly emetogenic cancer chemotherapeutics [59]. In fact, they are one major component of the so-called “triple prophylactic antiemetic therapy”, along with one of the above-described 5-HT_3_ receptor antagonists plus dexamethasone [50,51,52,60]. Basic studies have indicated that NK_1_R antagonists behave as broad-spectrum antiemetic [61] and are also effective against postoperative nausea and vomiting in the clinic [62,63]; however, they are not recommended in patients on birth control medications, since aprepitant reduces the effectiveness of oral contraceptives [59].

### 3.3. Dopamine D_2/3_ Receptors

Dopamine is a monoamine proemetic neurotransmitter and produces its diverse physiological effects through activation of two classes of membrane-bound G-protein-coupled receptors, namely dopamine D_1_-like (D_1_ and D_5_) and dopamine D_2_-like (D2, D3, and D4) receptors [64]. Surfeits or deficits in dopamine tissue levels results in several pathological conditions, including schizophrenia, Parkinson’s disease, attention deficit–hyperactivity, depression, and restless leg syndrome. Several dopamine-based agonists developed for the treatment of some of these diseases evoke nausea and vomiting as common impending adverse effects [65]. The discovery of role of dopamine in emesis began over seven decades ago on the proemetic effect of the nonselective dopamine receptor agonist apomorphine [66]. To date, numerous animal studies have implicated both dopamine D_2_ and D_3_ receptors in the mediation of vomiting [65]. In fact, nonselective (e.g., apomorphine) and dopamine D_2_ (e.g., quinpirole) and/or D_3_ (e.g., PNU95666E, 7-OH-DPAT) receptors preferring agonists elicit emesis in vomit-competent species, which can be attenuated by their corresponding competitive antagonists, whereas dopamine D_1_/D_4_/D_5_ receptor-selective agonists are devoid of emetic properties [67,68,69,70]. 

Dopamine, its proemetic receptors (dopamine D_2/3_), and/or their mRNA are well distributed in the emetic reflex arc, including the dorsal vagal complex (area postrema, nucleus of the solitary tract, dorsal motor nucleus of the vagus), vagal nerves, gastrointestinal tract, and the enteric nervous system [65]. In fact, dopamine tissue levels and turnover increase in the least shrew brainstem and jejunum during peak early and delayed phases of vomiting following cisplatin administration in the least shrew model of emesis [71]. Currently, several classes of dopamine D_2_-like receptor antagonists such as phenothiazines (e.g., chlorpromazine), butyrophenones (e.g., haloperidol), and benzamides (e.g., metoclopramide, which also blocks 5-HT_3_ receptors) are employed in the clinic for prevention of vomiting caused by diverse emetic stimuli, including: uremia, radiation sickness, viral gastroenteritis, postoperative nausea, and vomiting, as well as prophylaxis for cancer patients receiving chemotherapy with low emetogenic potential, or as a recue antiemetic in patients who have breakthrough emesis [65,72].

### 3.4. Acetylcholine Receptors

Acetylcholine plays important physiological functions and is a proemetic neurotransmitter. It activates the ligand-gated nicotinic receptor ion channels, as well as the G-protein-coupled muscarinic receptors consisting of five subtypes, M_1_–M_5_ [73,74]. Clinically used drugs that prevent metabolism of acetylcholine such as choline esterase inhibitors (e.g., donepezil, galantamine, rivastigmine, etc.) evoke vomiting both in humans and other vomit-competent species [75]. Direct injection of nicotine can evoke vomiting in cats [76]. Stimulation of muscarinic receptors also leads to vomiting; however, the precise role of each of the five muscarinic subtypes in vomiting remains to be fully defined. Muscarinic M_1_ receptor-evoked emesis have been demonstrated in animals. In fact, intracerebroventricular injection of the M_1_ receptor preferring agonist McN-A-343 causes dose-dependent vomiting in cats, which was prevented through ablation of the area postrema [77]. Moreover, systemic injection of McN-A-343 in least shrews also evokes vomiting [78]. In addition, more selective M_1_ receptor ligands are also emetogenic [79,80]. Currently available but nonselective muscarinic receptor antagonists such as scopolamine and atropine that also block M_1_ receptors are employed for the prevention of nausea and vomiting caused by motion sickness [81]. Transdermal scopolamine is also effective against postoperative nausea and vomiting [79,80].

### 3.5. Histamine H_1_ Receptor

The physiological effects of histamine are mediated via four histamine receptors, including H_1_, H_2_, H_3_, and H_4_ [82]. Significant preclinical evidence indicates that vestibular hyperactivity triggers activation of histaminergic neuronal system during motion sickness, which ultimately stimulates histamine H_1_ receptors in the brainstem to evoke vomiting [83]. In fact, intracerebroventricular injections of histamine causes vomiting, which was abolished by bilateral ablation of area postrema or pretreatment with antihistamines [84]. In addition, inhibition of histamine N-methyltransferase (HNMT), the enzyme responsible for metabolism of histamine with tacrine, increases endogenous histamine and exacerbates motion sickness in dogs, as well as susceptibility to condition taste aversion (an index of motion sickness) in rats [83]. Concomitantly, conditioned taste aversion was associated with histamine N-methyltransferase expression. Moreover, the latter authors have also demonstrated that, in rats: (i) microinjection of the H_1_ receptor antagonist promethazine to their dorsal vagal complex reduced condition taste aversion; (ii) repeated exposure to rotary stimulation habituated animals to motion sickness and elevated histamine N-methyltransferase expression in their brainstem; and (iii) elevation of histamine N-methyltransferase gene expression in the dorsal vagal complex inhibited the motion sickness, whereas introduction of recombinant lentiviral vectors expressing rat histamine N-methyltransferase-shRNA into the DVC to reduce the expression of histamine N-methyltransferase promoted motion sickness. Additionally, increased histamine tissue levels in the hypothalamus and brainstem nuclei such as the vestibular nucleus and the dorsal vagal complex are considered the primary reason for induction of motion sickness [83,85,86,87,88,89]. Although, like rats, mice do not vomit, motion sickness experience evokes increased levels of histamine, histamine H_1_ receptor mRNA, and H_1_ receptor protein expression in their hypothalamus and brainstem, which can be significantly reduced by pretreatment with hesperidin [83], a bioflavonoid that inhibits the synthesis and release of histamine from mast cells [90]. Histamine H_1_ receptor blockers such as dimenhydrinate and diphenhydramine are commonly used antiemetic agents against nausea and vomiting secondary to motion sickness [91,92,93]. It is important to note that many H_1_ receptor blockers have anticholinergic properties that block muscarinic receptors, which may also contribute to their antiemetic effects [81]. Histamine also has a peripheral emetic component, since its release from intestinal mast cells contributes to emesis in house musk shrews in response to food poisoning caused by Staphylococcal enterotoxins, to be discussed later (Section 4.6.1). Staphylococcal enterotoxins bind to jejunal submucosal mast cells and induce mast cell degranulation as well as the histamine release, which evokes vomiting through stimulating the vagal afferents and transmission to the brainstem emetic nuclei [94]. 

### 3.6. Opiate Receptors

Though the exact physiological mechanisms underpinning opioids-induced nausea and vomiting are not fully understood, activation of opiate receptors in the area postrema, vestibular apparatus and the gastrointestinal tract are implicated [14]. It appears all three opiate receptors (µ [mu], κ [kappa] and δ [delta]) may play a role in opioid-induced nausea and vomiting, but several studies indicate that activation of µ receptors is essential in emesis [95]. Expression of µ opioid receptor has been confirmed in the area postrema, nucleus of the solitary tract, vagal afferents, and the gastrointestinal tract [96,97]. The µ opioid receptor agonist loperamide has been shown to evoke emesis in the ferret, which could not be altered by abdominal vagotomy but was abolished by ablation of the area postrema, suggesting that vagal afferent input may not be important in µ opiate receptor-mediated emesis [98]. Opiate agonists may induce vomiting via multiple mechanisms: (i) direct stimulation of opioid µ (and probably delta receptors) and subsequent signaling to the nucleus of the solitary tract primarily via dopamine D_2_ receptors as well as 5-HT_3_ receptors present in the area postrema. (ii) Opioid µ or κ receptor agonists can inhibit intestinal motility that can lead to gut distension and increased emptying time, resulting in stimulation of visceral mechanoreceptors and chemoreceptors, which are also associated to nausea and vomiting. These emetic signals from the gastrointestinal tract to the brainstem nucleus of the solitary tract occurs through visceral afferents, which may further stimulate the already described serotonergic signaling pathway of vagal afferents [5]. (iii) Opioids can stimulate the vestibular apparatus, which is responsible for detecting changes in equilibrium, and sensory input to the nucleus of the solitary tract occurs via histamine H_1_ and cholinergic systems [13,14]. These inputs project to the nucleus of the solitary tract, which potentially has output pathways to the dorsal motor nucleus of the vagus to produce the vomiting reflex and projections to the mid- and forebrain for the perception of nausea [15]. Both emetic and antiemetic effects of µ opiate receptor agonists have been reported in animal studies [91,99]. There is some suggestion that, while µ opioid receptors located in the area postrema are involved in the mediation of emesis, those in the nucleus of the solitary tract provide inhibitory effects on vomiting [100], and genetic variants in the µ opioid gene also produce distinct effects on the nauseogenic and emetic potencies of opioids [92].

### 3.7. Neuropeptide Y2 Receptors

The neuropeptide Y hormone receptor family are activated by the three endogenous ligands: neuropeptide Y, peptide YY (PYY), and the pancreatic polypeptide processes [93,101,102]. Neuropeptide Y receptors consists of five G-protein coupled receptor subtypes (Y_1_, Y_2_, Y_4_, Y_5_, and Y_6_), which mediate a large variety of physiological and pathophysiological processes [93,101,102]. Following secretion by intestinal neuroendocrine L cells, peptide YY is hydrolyzed rapidly to PYY (3–36) [103]. PYY (1–36) and PYY (3–36) are both capable of crossing the blood brain barrier, with the latter being more active and abundant [104,105,106]. PYY (3–36) is a preferential agonist of the Y2 receptors [104]. 

In dogs and rats, high affinity binding sites for PYY are highly localized in the brainstem area postrema [107]. Peripherally, Y_2_ receptors have predominant neuronal distribution and are also localized on vagal afferent neurons [106] and myenteric and submucosal neurons [103]. Peptide YY (3–36) is a highly potent emetic peptide when given intravenously in dogs and bilateral lesion of the area postrema can prevent the evoked emesis [108]. In the mink, pretreatment with the selective brain-penetrant small molecule Y_2_ receptor antagonist JNJ-31020028 [109] has been shown to reduce PYY (3–36)-evoked vomiting [106]. In addition, peripherally administered peptide YY (3–36) exerts an anorectic effect through stimulation of Y_2_ receptors in the nucleus of the solitary tract (the terminal of the vagal afferents), while abdominal vagotomy can abolish the effect [110]. The anorectic effect has been used as an index of nausea in rodents [111]. The Y_2_ receptor agonists, PYY (3–36), and obinepitide cause nausea in humans [112,113,114]. 

Furthermore, the common foodborne mycotoxin deoxynivalenol triggers vomiting and elevates plasma levels of peptide YY(3–36) and 5-HT in the mink, which were inhibited by NPS-2143, an antagonist of the Ca^2+^-sensing receptor, or by ruthenium red, an antagonist of transient receptor ankyrin-1 [115]. 

### 3.8. GFRAL: Receptor of Growth Differentiation Factor 15 (GDF15)

GDF15 is a cytokine expressed in a variety of tissues and is secreted into circulation in response to diverse stimuli, targeting activation of hindbrain glial-derived neurotrophic factor receptor alpha-like (GFRAL), which has been identified as the GDF15 receptor [116]. Cisplatin induces higher circulating levels of GDF15 and activates GFRAL positive neurons in the area postrema/nucleus of the solitary tract regions of the dorsal vagal complex in the mouse brainstem [111]. Accumulating evidence support a direct role for GDF15 in the mediation of nausea and vomiting. In fact, GDF15 neutralization with monoclonal antibody attenuates cisplatin-induced emesis in nonhuman primates [117]. Elevated endogenous GDF15 has become a well-known marker associated with nausea and vomiting during pregnancy [118,119,120,121,122,123]. In addition, systemic administration of GDF15 causes emesis in musk shrews, and central or systemic GDNF injection can evoke nausea/emetic-like behaviors such as pica and kaolin consumption in nonemetic species [111]. 

### 3.9. Eicosanoid Receptors

Eicosanoid lipids, prostaglandins, and leukotrienes are arachidonic acid metabolites derived from cell membrane phospholipids, which are released by phospholipase A_2_ [124,125]. They are known as inflammatory mediators, but their role in the physiology and pathophysiology of the gastrointestinal tract is getting more attention [125,126]. Since they are chemically and metabolically unstable, after formation, they are released from cells immediately and usually function locally through specific G protein-coupled receptors on target cells [127]. 

#### 3.9.1. Prostaglandins and Receptors

The major types of prostaglandins include prostaglandin I_2_ (PGI_2_), prostaglandin D_2_ (PGD_2_), prostaglandin E_2_ (PGE_2_), and prostaglandin F_2α_ (PGF_2α_) [125]. Among them, Prostaglandin E_2_ (PGE_2_) is the most abundant prostaglandin [128]. There are currently ten known prostaglandin receptor subtypes on various cell types, termed DP1-2, EP1-4, FP, IP1-2, and TP [127]. 

A direct relationship between prostaglandins and nausea and vomiting is being established. In humans, elevated serum PGE2 levels is associated with increased episodes of nausea and vomiting in pregnancy [129]. Intravenous infusion of PGE_2_ or PGF_2α_ for abortion cause nausea and vomiting in up to ~20% patients and is worse in patients already suffering from hyperemesis gravidarum, an extreme form of nausea and vomiting of pregnancy [130,131]. In animals, several prostaglandins are found to be potential emetogens. In fact, intraperitoneal administration of the prostanoid TP receptor agonist U46619 evokes emetic behavior in ferrets [132] and Suncus murinus (house musk shrew) [133,134], which was antagonized by the TP receptor antagonist vapiprost [134]. However, the DP receptor agonist BW245C was approximately 1000 times less potent than U46619, EP (prostaglandin E_2_, 17-phenyl-omega-trinor prostaglandin E_2_, misoprostol, and sulprostone), FP (prostaglandin F_2α_ and fluprostenol), and IP (iloprost and cicaprost) receptor agonists and failed to induce emesis in the Suncus murinus [134]. Inconsistently, prostaglandin E_2_ is able to induce emesis in ferrets [135]. Furthermore, in ferrets, sulprostone, a prostanoid EP3/1 receptor selective agonist [136], was also emetic, and bilateral abdominal vagotomy was unable to reduce sulprostone-induced emesis, indicating the emetic action of sulprostone probably involves central mechanisms and is independent of the abdominal vagal afferents [137]. Another study performed in ferrets actually found varying efficacies of agonists of prostanoid receptor subtypes EP (sulprostone, prostaglandin E_2_), TP (U46619), DP (BW245C), and FP (prostaglandin F2alpha), but the IP agonist cicaprost was not effective in evoking emesis [138]. In addition, several prostaglandins (PGE_2_, 20-hydroxyPGE_2_, and PGF_2α_) evoke robust vomiting in the least shrew model of vomiting [139]. These forementioned animal studies focusing on the emetic actions of various prostanoid receptor agonists suggest the efficacy of different prostanoid receptor subtypes vary in mediating nausea and emesis. 

Corticosteroids block the biosynthesis of prostaglandins by inhibiting the phospholipase A2 and cyclooxygenase enzymes [140] and help to attenuate both the immediate and/or delayed phases of cisplatin-induced emesis in animals [141] and humans [142]. Besides their anti-inflammatory effects, glucocorticoids may act via the following mechanisms: (i) direct central action at the solitary tract nucleus; (ii) interaction with the neurotransmitter serotonin and receptor proteins of tachykinin NK_1_ and NK_2_, adrenaline, etc.; (iii) maintaining the normal physiological functions of organs and systems; and (iv) regulation of the hypothalamic–pituitary–adrenal axis [143]. 

#### 3.9.2. Cysteinyl Leukotrienes and Receptors

Of eicosanoids, the emetic role of the leukotriene family has been neglected. In least shrews, cysteinyl leukotrienes (LTC4, LTD4, and LTE4) but not its precursor leukotrienes (LTA4, LTB4, LTF4) induce vomiting with the different emetogenic efficacy (LTC4 = LTD4 > LTE4) [144]. LTC4-induced vomiting (i.p.) was prevented by the leukotriene CysLT1 receptor antagonist pranlukast but not by the mixed leukotriene CysLT1/2 receptor antagonist Bay u9773, suggesting that leukotriene CysLT1 receptor has a key role in emesis [144]. Montelukast, another classical CysLT1 antagonist [145], effectively blocks sevoflurane anesthesia-induced pica (Kaolin intakes) in rats [146]. In addition, in both intact and epithelium abraded guinea pig tracheal smooth muscle preparations, LTD4 causes substance P release [147]. 

## 4. Physiological Mechanisms of Emesis and Clinical Uses of Antiemetics

### 4.1. Chemotherapy-Induced Nausea and Vomiting (CINV) 

The platinum-based chemotherapeutic agents such as cisplatin, carboplatin, and oxaliplatin are regularly prescribed in the treatment of cancers [148]. Nausea and vomiting are common impending side effects of cancer cytotoxic chemotherapeutics [149]. As discussed earlier, the emetic action of these cytotoxic drugs initiates within the gastrointestinal tract, resulting in histopathological changes, increased biosynthesis of emetic neurotransmitters and their release from enterochromaffin cells and/or other tissue, with consequent increase in their intestinal tissue concentration [150]. In fact, cisplatin induces emesis via release of multiple classic emetic neurotransmitters such as 5-HT, substance P, and dopamine from the enterochromaffin cells and/or sensory neurons of the gastrointestinal tract, as well as the central nervous system during both acute and delayed phases of CINV [151]. The less well investigated emetic mediators in CINV are prostaglandins [130,131], leukotrienes [17,152], peptide YY [153], GDF15 [117], and oxidative stress [154]. 

*Prevention:* Various antiemetic guidelines have been developed for the prophylactic treatment of chemotherapy- and radiotherapy-induced nausea and emesis [50,51,52,60]. As discussed earlier, while the triple antiemetic therapy (a 5-HT_3_ receptor antagonist with an NK_1_ receptor antagonist plus the corticosteroid dexamethasone) has revolutionized prophylactic antiemetic therapy against both the acute and delayed phases of vomiting caused by highly emetogenic chemotherapeutic regimens, neither complete emesis protection in all cancer patients, nor significant nausea protection has yet been achieved [155,156].

### 4.2. Radiation-Induced Nausea and Vomiting 

Radiation-induced nausea and vomiting (RINV) are also common side effects of radiotherapy, which can affect up to 50–80% of patients [157,158]. While the mechanisms underlying RINV are relatively less well understood than CINV, the neurotransmitter basis and the processes responsible for “acute and delayed phases of RINV” are regarded as similar to CINV [159]. However, to the best of our knowledge, no investigation in either vomit-competent animals or humans has been conducted to characterize the validity of the two phases of RINV in a dose- and time-dependent fashion. In fact, the duration of acute RINV phase has arbitrarily been defined as the first day of radiotherapy to the first day after radiation exposure, while the second phase is regarded as nausea and vomiting occurring from day 2 up to 10 days posttherapy [160,161]. Vomiting can be initiated by release of emetic mediators when radiation therapy beams is directed toward the: (i) brainstem area or (ii) gastrointestinal portion of the body trunk, where splanchnic/vagal afferents release the aforementioned emetic neurotransmitter involved in CINV [162]. In fact, among the chemical mediators described in CINV, serotonin is the most studied emetic neurotransmitter pertaining to RINV [162], which makes 5-HT_3_ receptor antagonists the most commonly prescribed antiemetics for RINV prophylaxis [10]. In fact, in clinical trials, 5-HT_3_ receptor antagonists such as ondansetron, granisetron, and palonosetron appear to be effective in suppression both “acute” and “delayed” phases of RINV for both nausea (88–93% and 73–75%, respectively) and vomiting (93% and 75–93%, respectively) [160,161]. When granisetron was combined with the neurokinin NK_1_ receptor antagonist aprepitant, the antinausea (100 and 62%, respectively) and antiemetic (100 and 85%, respectively) effects were in a similar range during both phases [160,161].

### 4.3. Postoperative Nausea and Vomiting (PONV)

Postoperative nausea and vomiting and postdischarge nausea and vomiting (PDNV) remain common and distressing complications of surgery. The routine use of opioid analgesics for perioperative pain management is a major contributing factor to both PONV and PDNV after surgery [163]. Opioids, including morphine and fentanyl, are well-known inducers of nausea and vomiting as independent stimuli [164]. 

Clinical studies also indicate that PONV can be caused by inhalational anesthetics (e.g., sevoflurane, isoflurane, halothane), which appear to have equal potency for evoking PONV, as well as nitrous oxide (laughing gas) usage [15]. Nitrous oxide may contribute to PONV in several ways, including: (i) altering dopamine and opioid receptors function in the brain, (ii) producing changes in middle ear pressure, and/or (iii) causing bowel distension as it diffuses into closed cavities [164]. Isoflurane has been shown to increase c-Fos expression, a neuronal activation marker, in the area postrema of the rat, a vomit-incompetent species, which indicates activation of neurons in this brainstem region may be involved in the emetic actions of volatile anesthetics [165]. Increased vagal afferent activity can affect the vestibular system, which may also contribute to nausea and vomiting caused by inhalational anesthetics [15]. Inhalational anesthetics can enhance serotonin 5-HT_3_ receptor function in vitro, which could also contribute to nausea and vomiting [166]. Significant increases in the plasma concentrations of substance P has also been found in patients undergoing general anesthesia and postoperative nausea who exhibited vomiting, thus suggesting that substance P levels may be considered as an objective marker for PONV [167]. 

Prevention: The NK_1_ receptor antagonist aprepitant exerts improved antiemetic efficacy against both acute and delayed PONV, when compared with the 5-HT_3_ receptor antagonist ondansetron [168]. In patients with moderate to high risk of developing PONV, a combination of prophylactic antiemetic drugs with different mechanisms of action has been recommended [163]. In fact, prophylaxis, with the combination of a dopamine D_2_/_3_ receptor antagonist droperidol, corticosteroid dexamethasone, and a 5-HT_3_ receptor antagonist ondansetron, in conjunction with an NK_1_ receptor antagonist aprepitant, significantly reduces cumulative episodes of emesis through 48 h postoperatively, compared with discussed triple therapy prophylaxis, alone [169].

### 4.4. Cannabinoid Hyperemesis Syndrome

The cannabis plant (marijuana) and its main psychoactive component, delta-9-tetrahydrocannabinol (D^9^-THC), have been used as a second line prophylactic antiemetic medication for patients receiving cancer chemotherapeutics [170]. In fact, synthetic D^9^-THC (dronabinol) and nabilone (cesamet) have been approved by the FDA for this purpose. Unlike most antiemetics, cannabinoid agonists are classified as agonist antiemetics, and their broadspectrum antiemetic efficacy is mediated via activation of cannabinoid CB_1_ receptors [171]. Indeed, D^9^-THC produces its antiemetic effects via activation of both central and peripheral CB_1_ receptors in the brainstem dorsal vagal complex and the gastrointestinal tract [172]. Animal and clinical studies indicate that D^9^-THC can attenuate both the acute and delayed phases of chemotherapy-evoked vomiting [173]. Moreover, D^9^-THC and related cannabinoid agonists dose-dependently suppress vomiting caused by diverse emetogens, including the selective agonists of serotonin 5-HT_3_ [172], dopamine D_2_ [67], and neurokinin NK_1_ [174] receptor agonists, which are the major emetic receptor systems involved in both acute and delayed phases of chemotherapy-evoked vomiting [17]. Although D^9^-THC and other cannabinoids may have better antiemetic efficacy than dopamine D_2_ receptor antagonist antiemetics (such as prochlorperazine, chlorpromazine, haloperidol, or metoclopramide) [17], addition of D^9^-THC with 5-HT_3_ antagonists such as tropisetron or ondansetron do not substantially improve D^9^-THC’s antiemetic efficacy in either animals [17] or patients [175,176]. 

However, use of large doses of the cannabis plant or related synthetic cannabinoids on a long-term basis can cause a paradoxical hyperemetic effect known as cannabinoid hyperemesis syndrome (CHS). The CHS is characterized by cyclical episodes of nausea and vomiting [177,178]. Prolonged use of high doses of D^9^-THC or related cannabinoids result in changes to the endocannabinoid system which can dysregulate stress and anxiety responses, thermoregulation, the transient receptor potential vanilloid system, and several neurotransmitters systems, which could be potential mechanisms for induction of CHS [179]. 

Prevention: Typical antiemetics appear to be ineffective for the treatment of CHS, but anxiolytic and sedative drugs such as benzodiazepines (e.g., lorazepam, alprazolam), along with hot showers (or hot baths), seem to be consistently useful in reducing CHS symptoms [179]. Some studies have shown the dopamine D_2/3_ receptor antagonists such as haloperidol is also effective in treating acute CHS symptoms [180]. The hot component of chili powder capsaicin binds to the transient receptor potential vanilloid 1 (TRPV1) receptor and is also reported to be effective for acute treatment, while tricyclic antidepressants (e.g., amitriptyline) are considered for long-term CHS treatment [171]. Moreover, TRPV1 receptor agonists are another class of broad-spectrum antiemetics, and their ultrapotent agonist resiniferatoxin (RTX), at lower doses, has pro-, and at larger doses, antiemetic effects in ferrets, house musk shrews, and least shrews [181,182,183]. RTX appears to evoke emesis via release of substance P, perhaps with a co-transmitter, such as glutamate, at the nucleus of the solitary tract [182]. RTX has been shown to suppress vomiting in ferrets at a dose of 100 μg/kg (s.c.) when evoked by intragastric copper sulphate, total body X-radiation, or loperamide [181]. RTX can also attenuate vomiting in house musk shrews evoked by RTX itself, cisplatin, motion, and nicotine [182]. In the least shrew model of emesis, RTX at nanomolar non-emetic doses suppresses vomiting evoked by a diverse group of receptor specific emetogens, including selective and nonselective agonists of 5-HT_3_ receptor (5-HT and 2-Methyl-5-HT), dopaminergic D_2/3_ receptor (apomorphine and quinpirole), and cholinergic M_1_ receptor (pilocarpine and McN-A-343), as well as the selective neurokinin NK_1_ receptor agonist GR73632, the selective L-Type Ca^2+^ channel agonist FPL64176 and the sarcoplasmic endoplasmic reticulum calcium ATPase (SERCA) inhibitor thapsigargin [183]. The broad-spectrum antiemetic effects of RTX against stimuli acting via both central and peripheral inputs contribute to depletion of substance P at central emetic sites, as well as its ability in suppression of emetic transmitter release via inhibition of voltage activated Ca^2+^ channels, by increasing intracellular Ca^2+^ through vanilloid receptors [182]. 

TRPV1 receptors are found widely throughout the body, often in proximity to cannabinoid CB_1_ receptors in the dorsal vagal complex and gastrointestinal tract [171]. In fact, TRPV1 immunoreactivity is largely restricted to the nucleus of the solitary tract of the ferret, with faint labeling in the dorsal motor nucleus of the vagus and sparse distribution in the area postrema [184]. Cannabinoid CB_1_ receptors also have a similar distribution and are extensively colocalized with TRPV1 receptors, thus suggesting a functional interaction [184]. Indeed, a combination of CB_1_ and TRPV1 receptor agonists have the capacity to completely abolish cisplatin-induced emesis in least shrews at doses that are ineffective when used individually [185]. The TRPV1 receptor can be activated by marijuana, capsaicin, and heat, and chronic cannabis use inactivates TRPV1 receptors and alters gastric motility [186]. Exposure to nociceptive heat such as with compulsive hot-water bathing may transiently augment cutaneous TRPV1 firing and restore gastric motility, temporarily mitigating symptoms; therefore, hot water hydrotherapy is a mainstay of self-treatment for CHS [187]. By binding to TRPV1 receptors, topical capsaicin decreases the release of substance P from nerve endings, thereby decreasing nausea and vomiting in CHS [186]. Downregulation of NK_1_ receptor expression and receptor internalization also occurs in rat tissues following systemic capsaicin treatment [188]. The substance P neurokinin NK_1_ receptor antagonist class of antiemetics possess broad-spectrum antiemetic efficacy, and, as such, aprepitant has been used successfully in the treatment of CHS when all other common antiemetics failed [189]. Cessation of marijuana use gradually leads to normalization of TRPV1 function and can fully ameliorate CHS symptoms [186].

### 4.5. Motion Sickness: Mechanisms and Treatment 

Motion sickness is a common physiological response to real or virtual motion. Motion sickness occurs in both humans and animals during travel by sea, automobile, airplane, and in space, as well as under some other situations such as simulators, the cinema and video games [190]. Structures involved in motion sickness-induced nausea and vomiting have been well reviewed and studied [4,93,191,192], including the brainstem nucleus of the solitary tract, vestibular apparatus, cerebellum, hypothalamus, and visceral afferents. Therapy is directed toward decreasing conflicting sensory input, which is the most widely accepted theory for motion sickness, and as was discussed in Section 3.4 and Section 3.5. Antimuscarinics (e.g., scopolamine or atropine) and antihistamine H_1_ receptor antagonists (e.g., dimenhydrinate or diphenhydramine) are the most effective drugs [190] against motion sickness. Relative to the widely discussed physiological knowledge concerning motion sickness-induced nausea and vomiting, the basic molecular signaling mechanisms are not sufficiently investigated. Interestingly, studies using the mouse motion sickness model demonstrate that the increased TRPV1 protein level and phosphorylation of PI3K, AKT, mTOR, ERK, p38, and JNK are linked to motion sickness, while electro acupuncturing or deleting the TRPV1 gene in mice alleviated the aforementioned events, suggesting TRPV1 receptor and phosphoinositide 3-kinase (PI3K), protein kinase B (Akt), mammalian target of rapamycin (mTOR), extracellular signal-regulated kinase (ERK), p38 mitogen-activated protein kinase (MAKP), and c-Jun N-terminal Kinase (JNK) play important roles in the signaling mechanisms of motion sickness [193]. 

### 4.6. Emesis Induced by Microbial Infections:

Bacteria, viruses, and even fungi are often regarded as primitive organisms and are classically considered as individualistic, solely focused on self-preservation and proliferation. However, recent research has shed light on innumerous intrinsic processes, illustrating how these microorganisms exhibit sophisticated intracellular and signaling behaviors. Only in the last few decades was it even considered feasible that bacteria, viruses, and fungi could exhibit commensalism with a human host or delay their pathological properties until conditions are deemed adequate [194]. Specifically, bacteria possess the ability to communicate amongst themselves and with their hosts through means of various chemical signals, exquisitely represented through bacterial quorum sensing [195,196]. Here, we review the microbial intercellular communication responsible for emesis, illuminating these methods explicitly for the implication of therapeutic strategies against these processes. 

#### 4.6.1. Bacterial Infections and Emesis

Two of the most notable bacterial species responsible for emesis are unquestionably *Staphylococcus aureus* and *Bacillus cereus*. *Staphylococcus aureus (Staph aureus)* induces vomiting following consumption of their enterotoxins, producing what is known as food poisoning. Specifically, *Staph aureus* is known to possess staphylococcal enterotoxins, which are believed to function as in vivo immune response inhibitors [197,198]. Some of these staphylococcal enterotoxins can evoke vomiting [199]. In fact, intragastric or intraperitoneal administration of such staphylococcal enterotoxins can evoke vomiting in monkeys, dogs, piglets, and house musk shrews [199]. It appears that submucosal mast cells, enterochromaffin cells, and/or neurons present in the gastrointestinal tract are important targets for staphylococcal enterotoxins to bind, yet an unidentified receptor releases 5-HT in the house musk shrew small intestine. Indeed, the peripherally acting 5-HT synthesis inhibitor, p-chlorophenylalanine, or the 5-HT_3_ receptor antagonist granisetron, or surgical vagotomy, can suppress SEA-induced vomiting in house musk shrews [200]. Furthermore, staphylococcal enterotoxins can increase the cytosolic levels of Ca^2+^ via release of intracellular stored Ca^2+^ from the endoplasmic reticulum, which has been shown to be sensitive to nitric oxide synthase inhibitors [201]. The Ca^2+^-dependent release of 5-HT in the gastrointestinal tract is thought to stimulate vagal afferent serotonergic 5-HT_3_ receptors, which subsequently activates the dorsal vagal complex emetic nuclei in the brainstem to evoke vomiting [199].

*Bacillus cereus (B. cereus)*, most known for causing vomiting and diarrhea after eating reheated rice or other starchy foods, induces emesis by means of a heat stable peptide known as cereulide [191,202]. While diarrhea is caused by multicomponent toxins called enterotoxins produced in situ after excessive proliferation of *B. cereus* in the small intestine, emesis can be evoked by ingestion of cereulide, which accumulates in contaminated foods [199]. The reason *B. cereus* produces such similar symptomology to *Staph aureus* food poisoning is because the evoked mechanism responsible is believed to be very similar, though provoked by cereulide. Cereulide is a potassium ionophore and is structurally like valinomycin [192]. Like valinomycin and *Staph aureus* cereulide can activate vagal afferents [203]. Cereulide is stable and resists metabolism in the gastrointestinal tract and can induce vomiting in house musk shrews [203] and rhesus monkeys [204]. The exact mechanism of emetic effect of cereulide remains to be deciphered; however, it is known that it either directly stimulates serotonergic 5-HT_3_ receptors present on vagal gastrointestinal afferents or indirectly activates these receptors following release of 5-HT from enterochromaffin cells [199]. In fact, the 5-HT_3_ receptor antagonist ondansetron, or vagotomy, prevents the emetic effects of cereulide [203].

#### 4.6.2. Fungal Infections and Emesis

Fungi, on the other hand, induce vomiting by producing various mycotoxins, which range from generating only mild symptoms to causing fatality [205]. For instance, the mycotoxin deoxynivalenol, also known as vomitoxin, frequently infects foods of plant origin causing abdominal pain, nausea, vomiting, diarrhea, fever, dizziness, and headache when consumed. Deoxynivalenol is an organic, polar molecule mildly able to penetrate the blood brain barrier and increases the tissue levels of 5-HT and its metabolite 5-hydroxyindoleacetic acid in several regions of the brain, including the brainstem [206]. Additionally, deoxynivalenol also evokes Ca^2+^-dependent release of hormone which evokes emesis [115]. 

#### 4.6.3. Viral Signaling Mechanisms Responsible for Emesis

Lastly, we will review a few viral signaling mechanisms responsible for emesis. Viruses have developed their own ion channels to subvert their host cell’s basal ion-gradients to facilitate their replication and pathogenesis. As a result, viral proteins create holes in cellular membranes (i.e., viroporins such as nonstructural protein 4) through which ions and low-molecular-weight compounds can enter freely.

Rotaviruses: Ten different rotavirus (RV) species (A–J) have been classified and are a leading cause of severe, dehydrating gastroenteritis in children under the age of five. Rotavirus infections still cause >600,000 deaths per year despite global introduction of vaccinations [207]. As with some of the above discussed emetic mechanisms (e.g., chemotherapeutics, including cisplatin, and bacteria such as *S. aureus* and *Bacillus cereus*), RV infection also induces secretion of 5-HT from enterochromaffin cells, which plays a key role in vomiting after stimulation of vagal afferent nerves and activation of the dorsal vagal complex emetic nuclei in the brainstem. In fact, suppression of vomiting following rotavirus or noravirus infections with 5-HT_3_ receptor antagonists such as ondansetron supports the latter hypothesis [208].

The nonstructural protein (NSP4) of the RV enterotoxin has a major role in the induction of vomiting following rotavirus infection. Indeed, purified NSP4 has been shown to not only evoke release of serotonin from enterochromaffin cells but also increase intracellular Ca^2+^ concentration in a human midgut carcinoid enterochromaffin cell line in a dose- and time-dependent manner [208]. NSP4 has been thought to act as a viroporin by forming a cation channel in the endoplasmic reticulum, which is functionally responsible for increases in cytosolic Ca^2+^ levels [209]. Moreover, NSP4 mobilizes intracellular Ca^2+^ in human intestinal cells and in human intestinal enteroids by stimulating phospholipase C (PLC)-mediated inositol 1,4,5-trisphosphate (IP_3_) production and subsequent opening of IP_3_-sensitive calcium cannels in the endoplasmic reticulum membrane [210,211]. This leads to a reduction in endoplasmic Ca^2+^ stores, which activates stromal interaction protein 1, an endoplasmic calcium sensor, which, in turn, activates extracellular Ca^2+^ influx through store-operated calcium entry channels in the plasma membrane, primarily through Orai1 [210,211]. Thus, both intracellular calcium release and extracellular calcium influx are involved in NSP4-evoked emesis. In addition, RV infection in mice has been shown to evoke c-fos expression in the nucleus of the solitary tract, as seen in animals which vomit following administration of chemotherapeutic drugs [208,212].

Other viruses have also developed viroporins such as 2B/2BC (polio virus and coxsackievirus), human tlymphotropic virus 1, sindbis virus 6K, polyomavirus agnoprotein, influenza A (virus APB1-F2), and severe acute respiratory syndrome (SARS) Coronavirus (ORF3a), all of which modulate cytosolic Ca^2+^ levels [213]. Porcine delta-coronavirus has been shown to disrupt host Ca^2+^ homeostasis, which is believed to play a major role in the emetogenic reaction seen with infection of this virus [214]. This is because porcine delta-coronavirus’ Ca^2+^ mobilization is involved in the release of 5-HT via activation of the enteric nervous system and extrinsic vagal afferents to the brain, which leads to vomiting [214,215]. Similarly, the porcine epidemic diarrhea virus has been determined to evoke increased 5-HT release from enterochromaffin cells into the gut submucosa, thereby stimulating vagal afferent neurons and eliciting the emetic response [214,215]. 

Coronaviruses: Are a group of related RNA viruses that causes diseases in mammals and birds, including respiratory tract infections, neurological, and gastrointestinal disorders [216]. The first appearance of Coronavirus (CoV) occurred in 1960, which was considered nonvirulent but over four decades later underwent a drastic genomic change and caused SARS. Following further genetic alterations, it resurfaced again (MERS–CoV) in 2012 to cause the Middle East Respiratory Syndrome, and in 2019 appeared as the SARS–CoV-2, the novel coronavirus (2019–nCoV = Coronavirus Disease 2019 = COVID-19). While most COVID-19 patients typically present with fever and respiratory symptoms, depending upon study population, the prevalence of nausea and vomiting in COVID-19 patients can range from 2–26% and 2–20%, respectively [217]. The coronavirus is composed of a single-stranded RNA, and infection occurs by the binding of virions to the cellular receptors. Coronavirus infections are linked with deposition of the nucleocapsid into the cytoplasm, and, subsequently, the viral genome becomes ready for translation. Their structural proteins M, S, and E are inserted into the endoplasmic reticulum and passed to the Golgi apparatus. Eventually, vesicular virions are exported from the infected cells to the cellular membrane and are then released from these vesicles. Coronaviruses have spike protein, a glycoprotein that binds to the receptor of a host cell and induces viral entry. Their receptor binding domain is a small portion of the spike protein, which mediates binding with the host cell receptor. Following interaction with the receptor, host cell protease cleaves the spike and releases the fusion peptide, which facilitates virus entry into the host cell. The entry of MERS–CoV into the host cell conducted through the interaction of human dipeptidyl peptidase 4 and for SARS–CoV, through the interaction with Angiotensin-converting enzyme 2 receptor [216]. 

Based upon the mechanisms of emetic actions of cancer chemotherapeutic agents (cisplatin) and rotaviruses [207,209] discussed above, Andrews and co-workers (2021) have proposed that SARS–CoV-2-evoked emesis involves similar central and peripheral emetic mechanisms that are activated via local release of emetic mediators such as serotonin, substance P, and/or cholecystokinin from the upper intestinal mucosal enteroendocrine cells, which then activate vagal afferents, projecting to the dorsal vagal emetic nuclei in brainstem (e.g., area postrema, nucleus of the solitary tract, or dorsal motor nucleus of the vagus) and/or following entry into the blood to activate the area postrema of the brainstem. In addition to activation of vagal afferents, CoVs can access the brain via hematogenous and neurogenic mechanisms [218]. Hematogenous invasion can involve crossing the blood brain barrier or hijacking peripheral blood cells such as monocytes/macrophages, which get infected in the lungs. Neurogenic invasion could involve retrograde axonal transport along peripheral nerves such as the olfactory, trigeminus, and vagal nerves. The spike (S) protein is the main determinant of cell tropism and pathogenicity of SARS–CoV-2, which mediates its entry into host cells via its receptor, the angiotensin 2 converting enzyme [218]. Both angiotensin-converting enzyme 2 and the cellular serine protease transmembrane protease serine-2 are required for viral entry, which are expressed in upper gastrointestinal tract enterocytes. The angiotensin 2 converting enzyme is expressed on enteroendocrine cells, and SARS–CoV-2 infects enterocytes but not enteroendocrine cells (studies needed with native enteroendocrine cells) [219]. The subsequent virus-evoked release of epithelial emetic mediators via exocytosis, inflammation, and apoptosis may trigger the intestinal and brainstem emetic drives. Moreover, SARS–CoV-2 increases plasma angiotensin II levels, which is a centrally (area postrema) acting emetic agent, thus providing an additional mechanism for COVID-19-evoked emesis. Viral invasion of the dorsal brainstem can be also a possibility, but more likely during delayed onset symptoms [219]. 

#### 4.6.4. Microbiota

Among the various functions of gut microbiota is their ability to resist colonization of pathogenic bacteria [220]. Antibiotics are often generalized to kill both pathogenic and beneficial bacteria, which can alter the function and decrease the diversity of the gut microbiota [220]. Because of this, pathogenic bacteria can flourish and cause infection without resistance from the gut microbiota. Clostridium difficile is a bacterium that colonizes the large intestine and can cause clostridium difficile infection (CDI) when antibiotics deplete the protective intestinal microbiota [221,222]. Common symptoms associated with CDI are nausea, emesis, diarrhea, and abdomen distension [223]. This indicates that depletion of the protective intestinal microbiota may play a protective role against emesis. An effective treatment for severe CDI has been fecal microbiota transplantation (FMT) along with vancomycin to prevent the growth of the bacteria while replenishing lost microbiota [223]. Treating CDI with FMT further indicates that balancing microbiota can prevent emesis due to bacterial infection. 

As mentioned above, the SARS–Cov-2 virus is now known not only to affect the respiratory system but the gastrointestinal system, as well [224], leading to diarrhea and emesis, especially in the early phase of the disease [225]. The virus’s effect of emesis may be due to the way the gut microbiome is altered. Patients infected with SARS–Cov-2 have an altered microbiome due to alteration of B and T cells in the intestines and activation of the enteric system. Destruction of the microbiota comes secondary to inflammation of the gut due to viral infection. It has been reported that more than half of the COVID-19 patients in Zhejiang, China, had microbial dysbiosis and tested positive for SARS–Cov-2 RNA in their feces [226]. In a smaller study, with 15 COVID-19 patients, authors found a positive correlation between COVID-19 severity and the bacteria Coprobacillus, Clostridium ramosum, and Clostridium hathewayi, and a negative correlation between COVID-19 severity and the anti-inflammatory bacterium Faecalibacterium prausnitzii [227]. These changes in the gut microbiota during SARS–Cov-2 infection could be correlated to emesis seen during early disease.

## 5. Intracellular Signal Transduction Systems in Emesis

### 5.1. Calcium

Per our previous reviews [228,229], among the diverse receptor-mediated intracellular emetic signaling cascades, one potential converging signal appears to be changes in the cytosolic concentration of calcium (Ca^2+^). Cytoplasmic Ca^2+^ concentration is the dominant element in determining the amount of transmitter to be released from nerve terminals [230]. Thus, Ca^2+^ mobilization can be an important aspect of vomit induction, since it is involved in both triggering the quantity of neurotransmitter released coupled with receptor activation, as well as postreceptor excitation–contraction coupling mechanisms [231]. Studies using Ca^2+^ imaging performed in vitro in the brainstem slice preparation suggest that emetic agents evoke direct excitatory effects on cytosolic Ca^2+^ signals in vagal afferent terminals in the nucleus of the solitary tract which potentiate local neurotransmitter release [232,233,234]. 

#### 5.1.1. Emetogens Mobilize Ca^2+^

Receptor activation by corresponding agonists can increase cytosolic Ca^2+^ levels both via mobilization of intracellular Ca^2+^ stores from the endoplasmic reticulum and extracellular Ca^2+^ influx resulting from direct opening of diverse ion channels present on the cell membrane or indirectly via signal transduction pathways following G protein-coupled receptor activation [235]. The serotonin 5-HT_3_ receptor is a Ca^2+^-permeable ligand-gated ion channel [236]. Cell line studies have demonstrated that stimulation of 5-HT_3_ receptors can evoke an influx of extracellular Ca^2+^ that is sensitive to selective antagonists of both 5-HT_3_ receptors (e.g., tropisetron, MDL7222, metoclopramide) and L-type calcium channels (LTCCs) such as verapamil, nimodipine, or nitrendipine [236,237,238,239]. Thus, it appears that both LTCCs and 5-HT_3_ receptor Ca^2+^-permeable ion channels are involved in extracellular Ca^2+^ influx induced by 5-HT_3_ receptor activation. Moreover, 5-HT_3_ receptor stimulation and the evoked extracellular Ca^2+^ entry trigger release of intracellular Ca^2+^ from ryanodine-sensitive sarco/endoplasmic reticulum calcium stores via a process often referred to as Ca^2+^-induced Ca^2+^ release [238], which greatly amplifies the cytosolic concentration of Ca^2+^ [238]. Although activation of the substance P neurokinin NK_1_ receptors also increases the cytosolic levels of calcium, it does so via different signaling mechanisms, including an initial intracellular Ca^2+^ release to deplete intracellular stored calcium from the sarco/endoplasmic reticulum through activation of the phospholipase C (PLC)/inositol trisphosphate pathway (see later) and subsequent extracellular calcium influx through plasma–membrane-bound calcium channels, including store-operated Ca^2+^ entry and LTCCs [240,241,242,243]. In fact, the NK_1_ receptor selective agonist GR73632 induces vomiting in least shrews by an initial depletion of stored sarco/endoplasmic calcium and subsequent extracellular Ca^2+^ influx mainly through opening of LTCCs and, to a smaller extent, via SOCE [244]. Other emetogens such as agonists of dopamine D_2_ [245,246], cholinergic M_1_ [247,248], histaminergic H_1_ [240,249] and mu opiate [250,251] receptors, as well as cisplatin [241,242,252,253], prostaglandins [243,254], rotavirus NSP4 protein [208,209], fungal toxins [115], and bacterial toxins [255,256], also possess the potential to mobilize Ca^2+^ that involve extracellular Ca^2+^ influx and/or Ca^2+^ release from intracellular pools. 

#### 5.1.2. Pro- and Antiemetic Effects of L-Type Calcium Channels (LTCC) Agonists and Antagonists 

LTCCs serve as the principal route of Ca^2+^ entry in electrically excitable cells such as neurons and muscle [257,258]. FPL64176 is a selective agonist of LTCCs and causes vomiting in least shrews in a dose-dependent and LTCC antagonist-sensitive manner [78,244]. In addition, all tested shrews vomit at a 10 mg/kg dose of FPL64176 administered intraperitoneally (i.p.) [78,244]. These findings directly support our notion that Ca^2+^ mobilization is an important facet in the mediation of emesis. In fact, LTCCs have been shown to be present on the enterochromaffin cells of guinea pig and human small intestinal crypts [259] and increased cytoplasmic Ca^2+^ evoked by emetic stimuli such as radiation or FPL64176 evoke release of emetic neurotransmitters such as serotonin from these cells [260].

Blockade of extracellular Ca^2+^ entry through LTCCs cause inhibition of excitation-coupled contraction [261]. In the least shrew emesis model, the LTCC blockers nifedipine and amlodipine suppress vomiting in a potent and dose-dependent fashion when evoked by diverse emetogens, including agonists of LTCC (FPL64176), serotonin 5-HT_3_ (e.g., 5-HT or 2-Methyl-5-HT), neurokinin NK_1_ (GR73632), dopamine D_2/3_ (apomorphine or quinpirole), and muscarinic M_1_ (McN-A-343) receptors [78]. In addition, intracerebroventricular microinjection of another LTCC antagonist nitrendipine can attenuate nicotine-induced vomiting in the cat [262]. Likewise, scant clinical case reports also demonstrate the antiemetic potential of LTCC antagonists, since flunarizine has been reported to reduce cyclic vomiting on an acute basis in one patient [263] and prophylactically in eight patients [264]. The mechanism underlying the broad-spectrum antiemetic potential of nifedipine against these diverse emetogens can be closely related to inhibition of Ca^2+^ mobilization and the emetic receptors-driven downstream signaling pathways involving key molecules [229].

#### 5.1.3. Involvement of Other Ca^2+^ Channel Modulators in Emesis

The sarco/endoplasmic reticulum Ca^2+^-ATPase (SERCA) pump transports the free cytosolic Ca^2+^ into the lumen of sarco/endoplasmic reticulum to counterbalance the cytosolic intracellular Ca^2+^ release from the sarco/endoplasmic reticulum into the cytoplasm via inositol 1,4,5-trisphosphate- (IP_3_Rs) and ryanodine receptors (RyRs) present on the wall of sarco/endoplasmic reticulum [265,266,267]. The intracellular Ca^2+^ mobilizing agent thapsigargin is a selective SERCA inhibitor, which increases cytosolic Ca^2+^ concentration via an initial intracellular release of stored Ca^2+^ from the sarco/endoplasmic reticulum into the cytosol, followed by store-operated extracellular Ca^2+^ entry (SOCE) [268,269,270]. In total, these events lead to a significant rise in the free concentration of cytosolic Ca^2+^ [271,272,273]. In least shrews, intraperitoneal administration of thapsigargin (0.1–10 mg/kg) causes vomiting in a bell-shaped manner, with maximal efficacy at 0.5 mg/kg [274]. 

The role of RyRs- and IP_3_Rs-mediated intracellular Ca^2+^-release through pharmacological use of their respective antagonists dantrolene and 2-APB in vomiting has been carried out in our laboratory, and the attained results help to support the involvement of these intracellular Ca^2+^ release channels in emesis. In fact, in the least shrew emesis model, we have demonstrated vomiting evoked by either the 5-HT_3_R agonist 2-methyl-5HT or the LTCC agonist FPL64176 were insensitive to 2-APB, but, in contrast, both the frequency and percentage of shrews vomiting were dose-dependently suppressed by dantrolene [244]. On the other hand, pretreatment with either dantrolene or 2-APB leads to significant reductions in the frequency of thapsigargin-induced vomiting [274]. Moreover, pretreatment with 2-APB causes a significant reduction in substance P neurokinin NK_1_R agonist GR73632-induced emesis; however, the RyR inhibitor dantrolene did not affect the evoked vomiting (Zhong, Chebolu et al., 2019). Thus, RyRs and IP_3_Rs can be differentially modulated by diverse emetogens, and suppression of Ca^2+^ release from sarco/endoplasmic reticulum through IP_3_Rs and RyRs may provide additional targets for suppression of nausea and vomiting. SOCE inhibitors such as MRS1845 also provide some antiemetic effect in such studies [244,274,275,276]. The potential antiemetic role of SOCE inhibitors needs to be further studied in vomiting evoked by other emetogens, since it mediates Ca^2+^ entry in the PLC-coupled GPCRs signaling.

### 5.2. Intracellular Emetic Signals in Diverse Signal Transduction Pathways 

Historically, studies focusing on intracellular emetic signaling mechanisms are limited to enhancement of cyclic adenosine monophosphate (cAMP) levels after inhibition of its metabolism by phosphodiesterase inhibitors [277]. However, more recently, significant advances in the field have been made using the least shrew emesis model in our laboratory, which are described below (Table 1):

#### 5.2.1. Adenylyl Cyclase (cAMP)–Protein Kinase A (PKA) 

The cAMP–PKA signaling pathway is a universal mediator of signal transduction that relays extracellular stimuli, including hormones or neurotransmitters, to intracellular targets. PKA is a downstream signal in the adenylyl cyclase/cAMP pathway that phosphorylates target proteins such as nuclear receptors and transcription factors, other kinases and phosphatases, G-protein coupled receptors, and ion channels [282]. The emetic role of adenylyl cyclase/cAMP/PKA pathway has been well established, since microinjection of cAMP analogs (e.g., 8-bromocAMP) or forskolin (to enhance endogenous levels of cAMP) in the brainstem dorsal vagal complex emetic locus area postrema not only increases electrical activity of local neurons but also induces vomiting in dogs [283]. Moreover, administration of 8-chlorocAMP in cancer patients can evoke nausea and vomiting [284]. Furthermore, phosphodiesterase inhibitors such as rolipram prevent cAMP metabolism and, consequently, increase cAMP tissue levels, which leads to excessive nausea and vomiting in humans [277]. In fact, one major side effect of older phosphodiesterase inhibitors is excessive nausea and vomiting, which often precludes their use in the clinical setting [285]. In addition, we have demonstrated that vomiting evoked via increased brainstem cAMP levels can be prevented by SQ22536, an inhibitor of adenylyl cyclase [281]. Moreover, PKA phosphorylation is associated with peak vomit frequency during both the immediate and delayed phases of vomiting caused by administration of either cisplatin or cyclophosphamide in the least shrew model of emesis [279,281,286]. Furthermore, enterochromaffin cells release serotonin via a PKA-dependent mechanism [287].

#### 5.2.2. Extracellular Signal Regulated Kinase 1 and 2 (ERK1/2) 

ERK1/2 are members of the mitogen activated protein kinase superfamily, and their signaling pathway is implicated in a diverse array of cellular functions, including cell differentiation, proliferation, and inflammatory responses. ERK1/2 phosphorylation has been used to indicate neuronal activation [288]. Our published findings have implicated ERK1/2 signaling in the brainstem and/or jejunum as a common signaling factor in the regulation of emetic responses elicited by systemic administration (i.p.) of diverse emetogens, including the: (i) NK_1_ receptor agonist GR73632, ii) LTCC activator FPL64176, (iii) the SERCA inhibitor, a Ca^2+^ signaling amplifier, thapsigargin, (iv) the 5-HT_3_ receptor agonist 2-Methyl-5-HT [244,274,275,276], (v) chemotherapeutic agent cisplatin [279], and (vi) Akt inhibitor MK-2206 [280]. In addition, inhibitors of ERK1/2 prevent the evoked vomiting in the above studies.

#### 5.2.3. Ca^2+^/Calmodulin-Dependent Kinase II (CaMKII) 

CaMKII is a serine/threonine-specific protein kinase that is regulated by the Ca^2+^/calmodulin complex and consists of two types, CaMKIIα and CaMKIIb. Time-dependent CaMKIIα phosphorylation in the least shrew brainstem occurs following: (i) serotonin 5-HT_3_R-evoked vomiting mediated by its selective agonist 2-methyl-5-HT, (ii) thapsigargin-induced emesis, (iii) substance P neurokinin NK_1_R-induced vomiting evoked by its selective agonist GR73632, and (iv) vomiting caused by the selective LTCC agonist FPL64176 [254,289,290]. Pretreatment with selective inhibitors of CaMKII offers partial antiemetic efficacy in these studies. 

#### 5.2.4. Protein Kinase C (PKC)

PKC consists of a family of 10 serine/threonine kinases, which are dependent on phosphatidylserine, calcium, and diacylglycerol for activity (Black and Black 2021). Our published studies demonstrate that phosphorylation of protein kinase Cα/βII (PKCα/βII) in least shrew brainstem is associated with vomiting evoked by the: (i) chemotherapeutic agent, cisplatin [279,286], (ii) LTCC activator FPL64176, and (iii) substance P neurokinin NK_1_ receptor agonist, GR73632 [244,275]. The PKC inhibitor GF109203X given intraperitoneally can reduce vomiting evoked by the latter two emetogens [244,275].

#### 5.2.5. Akt/Glycogen Synthase Kinase 3 (GSK-3)

The Akt/GSK-3 pathway is a critical signaling pathways that is dysregulated in numerous human cancers. The Akt cascade acts in cancer process by regulating apoptosis, cell cycle, metabolism, and cells’ longevity. Akt phosphorylation has been observed in the least shrew brainstem following vomiting evoked by the: (i) substance P selective NK_1_ receptor agonist GR73632 and (ii) selective LTCC agonist FPL64176 [254,289,290]. We have recently demonstrated that the Akt inhibitors can evoke vomiting in a dose-dependent manner in least shrews, where perifosine caused emesis in 90% of tested shrews and MK-2206 in all tested animals [280]. In fact, nausea and vomiting are important impending side effects when cancer patients are treated with these Akt inhibitors [291,292].

The GSK-3 enzyme is a constitutively active multifunctional serine–threonine kinase that affects diverse physiological processes. In fact, GSK-3 has been linked in a broad range of diseases, including neurodegeneration, inflammation, diabetes, and cancer. GSK-3 is a downstream target for Akt, which phosphorylates GSK-3 and suppresses its activity [289,290,293,294,295,296,297]. Activation of Akt signaling can be followed by phosphorylation of GSK-3α/β at Ser21/9 and its subsequent inactivation [290]. We have recently demonstrated that GSK-3 plays an important role in vomiting [298]. In fact, in least shrews brainstem, phosphorylation of GSK-3α and GSK-3β subtypes display a time-dependent increase in response to systemic administration of a variety of emetogens, including agonists of 5-HT_3_ (e.g., 5-HT or 2-Methyl-5-HT), neurokinin NK_1_ (GR73632), dopamine D_2/3_ (apomorphine or quinpirole), and muscarinic M_1_ (MCN-A343 or pilocarpine) receptors, as well as the LTCC agonist FPL64176 and the SERCA inhibitor thapsigargin [298]. In addition, selective GSK-3 inhibitors such as AR-A014418 and SB216763 significantly suppress both the frequency and percentage of shrews vomiting evoked by the above specific emetogens. Furthermore, SB216763 behaved as a more potent antiemetic agent [298]. These data support an interesting hypothesis that, following pharmacological induction of vomiting, the evoked phosphorylation of Akt, along with the subsequent phosphorylation/inhibition of GSK-3, may exert a unique self-protective effect to avoid further vomiting. More details regarding the role of Akt and GSK-3 in emesis have been recently published [299]. 

## 6. Conclusions

The presence of the vomiting reflex is widespread among mammals, such as carnivores (e.g., cat, dog, ferret), primates (e.g., human, monkey), and insectivores (e.g., shrews) [39,300]. The lack of emetic responses in rodents, including rats and mice, has been attributed to differences in upper alimentary tract anatomy and brainstem neural circuitry [39,300]. Because of the lack of the vomiting reflex in these species, the suitability of such animals for the study of nausea using behavioral markers such as conditioned taste aversion and pica (clay ingestion) in responses to emetic agents has been problematic [39,300]. 

Although systematic research in the area of nausea and vomiting began in the late 1950s, the main impetus in this field of research has been development of receptor selective antiemetics such as the first- and second-generation serotonin 5-HT_3_ and substance P neurokinin NK_1_ receptor antagonists combined with the glucocorticoid dexamethasone, which protect up to 80–90% of cancer patients from vomiting who were prescribed highly emetogenic cytotoxic chemotherapeutic regimens such as cisplatin. Experimental use of selective receptor antagonist antiemetics has led to the current body of knowledge, but future development of antiemetics probably lies in development of drugs that target intracellular emetic signals. In addition, bacteria, viruses, and fungi enter cells by known and unknown mechanisms and hijack hosts’ intracellular signaling mechanisms to evoke vomiting, and drugs that block membrane-bound receptors may not be useful. With the multifaceted and comprehensive knowledge reviewed here, further studies are required to ascertain the “cross talks” between intracellular nauseogenic and emetogenic signaling pathways caused by diverse specific and nonspecific emetogens. Additional studies should involve potential antiemetic compounds targeting common or specific intracellular emetic signaling pathways, alone or in combination with conventional antiemetics of choice.

## Figures and Tables

**Table 1:** Intracellular signaling molecules affected by vomiting-evoked by diverse emetogens in the least shrew vomiting model. The emetics include selective receptor agonists and nonselective chemotherapeutics, e.g., cisplatin and cyclophosphamide.

Target Receptor/Protein	Emetogens (Agonist/Inhibitor)	Affected Signaling Molecules	References
Serotonin 5-HT_3_ receptor	2-Methyl-5-HT (agonist)	Phosphorylation of CaMKIIα and ERK	Zhong et al., 2014 [276], Hutchinson et al., 2015 [278]
Neuroknin NK_1_ receptor	GR73632 (agonist)	Phosphorylation of CaMKIIα, ERK, Akt, and PKC	Zhong et al., 2019 [275]
L-type Ca^2+^ channel	FPL64176 (agonist)	Phosphorylation of ERK1/2, PKC, and Akt	Zhong et al., 2018 [244]
Nonselective	Cisplatin	Phosphorylation of proteins ERK1/2, PKC, and PKA	Darmani et al., 2015 [279]
Sarcoplasmic endoplasmic reticulum calcium ATPase	Thapsigargin (inhibitor)	Phosphorylation of CaMKIIα and ERK	Zhong et al., 2016 [274]
Akt	MK-2206 (inhibitor) Perifosine (inhibitor)	Phosphorylation of ERK	Zhong et al., 2021 [280]
Nonselective	Cyclophosphamide	Tissue level of cAMP, phosphorylation of PKA	Alkam et al., 2014 [281]
Phosphodiesterase	Rolipram (inhibitor)	Tissue level of cAMP	Alkam et al., 2014 [281]

## Abbreviations

central nervous system (CNS), area postrema (AP), nucleus of the solitary tract (NTS), dorsal motor nucleus of the vagus (DMNV), serotonin (5-HT), substance P (SP), calcium (Ca^2+^), serotonin type 3 receptor (5-HT3 receptor), neurokinin-1 receptor (NK1 receptor), functional magnetic resonance imaging (fMRI), transient receptor potential vanilloid type 1 receptor (TRPV1), G-protein-coupled receptor (GPCR), intraperitoneally (i.p.), peptide YY (PYY), growth differentiation factor 15 (GDF15), glial-derived neurotrophic factor receptor alpha-like (GFRAL), prostaglandin (PG), chemotherapy-induced nausea and vomiting (CINV), postoperative nausea and vomiting (PONV), radiation-induced nausea and vomiting (RINV), delta-9-tetrahydrocannabinol (D^9^-THC), cannabinoid receptor 1 (CB1 receptor), cannabinoid hyperemesis syndrome (CHS), resiniferatoxin (RTX), sarcoplasmic/endoplasmic reticulum calcium ATPase (SERCA), phosphoinositide 3-kinase (PI3K), protein kinase B (Akt), extracellular signal-regulated kinase (ERK), c-Jun N-terminal Kinase (JNK), mitogen-activated protein kinase (MAPK), mammalian target of rapamycin (mTOR), rotavirus (RV), nonstructural protein (NSP4), phospholipase C (PLC), inositol 1,4,5-trisphosphate (IP3), severe acute respiratory syndrome (SARS), coronavirus (CoV), L-type Ca^2+^ channels (LTCC), inositol 1,4,5-trisphosphate receptor (IP_3_ receptor), ryanodine receptor (RyR), store operated Ca^2+^ entry (SOCE), cyclic adenosine monophosphate (cAMP), protein kinase A (PKA), calmodulin-dependent protein kinase II (CaMKII), protein kinase C (PKC), Glycogen Synthase Kinase-3 (GSK-3).
